# Fulminant *Fusobacterium necrophorum* Meningoencephalitis Secondary to Frontal Sinusitis in a Previously Healthy Young Adult: A Case Report

**DOI:** 10.3390/idr18050097

**Published:** 2026-09-02

**Authors:** Amir Hossein Pirasteh, Katrin Streubel, Sabine Wagner, Jorge Hugo Coello Alvarez, Christopher Nimsky, Sabrina Viktoria Kirchleitner

**Affiliations:** 1 Department of Neurosurgery, University Hospital Marburg, Philipps University Marburg, 35043 Marburg, Germany; jorgehugo.coelloalvarez@uk-gm.de (J.H.C.A.); christopher.nimsky@uk-gm.de (C.N.); sabrinaviktoria.kirchleitner@uk-gm.de (S.V.K.); 2 Department of Microbiology, University Hospital Marburg, Philipps University Marburg, 35043 Marburg, Germany; katrin.streubel@uk-gm.de; 3 Department of Neuroradiology, University Hospital Marburg, Philipps University Marburg, 35043 Marburg, Germany; sabinemarianne.wagner@uk-gm.de; 4 Center for Mind, Brain and Behavior (CMBB), 35043 Marburg, Germany

**Keywords:** *Fusobacterium necrophorum*, meningoencephalitis, sinusitis, intracranial empyema

## Abstract

Background: *Fusobacterium necrophorum* is an anaerobic Gram-negative organism classically associated with severe head and neck infections and Lemierre syndrome. Central nervous system involvement is uncommon but may be rapidly progressive and fatal, particularly when associated with sinusitis, intracranial empyema, cerebritis, or brain abscess. Case presentation: We report the case of a previously healthy 18-year-old male who presented after four days of high fever up to 41.5 °C, severe holocranial headache, vomiting, and acute hyperactive delirium. On admission, he had meningismus, markedly elevated inflammatory markers, and concomitant *Influenza A* infection. The initial cranial computer tomography showed a left frontal hypodense lesion with hemorrhagic transformation and perfusion deficit. The cranial magnetic resonance tomography demonstrated left frontopolar cerebritis, intracranial empyema along the falx and tentorium, intraspinal extension, and extensive bilateral frontal, maxillary, and ethmoidal sinusitis, suggesting sinogenic intracranial spread. The patient underwent emergency bilateral pansinus surgery, placement of an external ventricular drain, left hemicraniectomy with evacuation of empyema, repeat evacuation of subdural empyema and frontal abscess drainage, and posterior fossa decompression with C1 laminectomy. *Fusobacterium necrophorum* was detected in anaerobic blood cultures and the operative intracranial samples. Despite aggressive interdisciplinary management, the patient developed septic multiorgan failure and died on hospital day 4. Conclusions: This case illustrates a rare but potentially fatal sinogenic *Fusobacterium necrophorum* infection of the central nervous system in a young adult. By providing a detailed report, we intend to increase awareness of this rare yet fatal clinical presentation.

## 1. Introduction

*Fusobacterium necrophorum* is an anaerobic Gram-negative bacillus that may cause invasive infections in adolescents and young adults, most commonly in association with oropharyngeal or other head and neck infections [1]. Although the organism is well known in relation to Lemierre syndrome, central nervous system involvement is rare [1,2,3,4,5,6]. Reported CNS manifestations include meningitis, cerebritis, subdural empyema, cerebral abscess, venous thrombosis, hydrocephalus, and fatal intracranial hypertension [2,3,4,5,6,7,8,9]. Sinogenic intracranial infection represents a severe complication of bacterial sinusitis [7,8,10]. Clinical deterioration may be rapid, and early symptoms can overlap with viral illness or uncomplicated upper respiratory infection. Published experience with *F. necrophorum* meningitis and intracranial empyema remains limited, especially in immunocompetent young adults [2,3,4,5,6,7,8,9]. We present a fulminant fatal case of sinogenic *F. necrophorum* meningoencephalitis with cerebritis, intracranial empyema, brain abscess formation in a previously healthy 18-year-old male. The aim is to document the clinical course, diagnostic findings, microbiological results, surgical management, and outcome, and to raise awareness of this rare yet severe presentation.

## 2. Case Report

An 18-year-old male with no known comorbidities, no regular medication, and no known allergies was brought to the emergency department. He had developed a high febrile illness four days earlier, with temperatures reported up to 41.5 °C and severe holocranial headache. Vomiting had been present for two days. The day before admission, he had been assessed by an outpatient otorhinolaryngologist for suspected sinusitis and was prescribed decongestant nasal drops without antibiotics. On the morning of admission, he became increasingly confused and developed hyperactive delirium.

On arrival, the patient was awake but severely agitated and disoriented, with a retrospectively estimated GCS of 9–10. He was hemodynamically stable, with systolic blood pressure ranging from 110–140 mmHg and diastolic blood pressure from 60–90 mmHg. Respiratory rate was mildly elevated at 20–30/min in the context of marked agitation, and body temperature was 39.5 °C. He had no verbal output and did not follow simple commands. Pupils were round, equal, medium-sized, and reactive to light. No obvious paresis was documented; motor response to painful stimuli was present in all extremities. Reflexes were medium bilaterally, Babinski signs were negative, and meningismus was present.

Initial laboratory testing showed leukocytosis of 12.8 G/L, a CRP-value of 272.9 mg/L, and a procalcitonin-value of 25.0 µg/L. D-dimers were elevated at 8.27 mg/L. *Influenza A* testing was positive. The cerebrospinal fluid (CSF) analysis showed a pleocytosis with 9000 cells/mm^3^, a decreased glucose level (21 mg/dL) and an increased protein level (4.28 g/L). CSF Gram stain, culture, multiplex PCR, 16S bacterial PCR, fungal testing, and mycobacterial testing remained negative. Initial emergency treatment included intravenous ceftriaxone 2 g, dexamethasone 10 mg, and acyclovir 750 mg. Because of severe agitation and reduced vigilance, sedation and endotracheal intubation were required to complete diagnostic imaging and further treatment.

**Figure 1:**
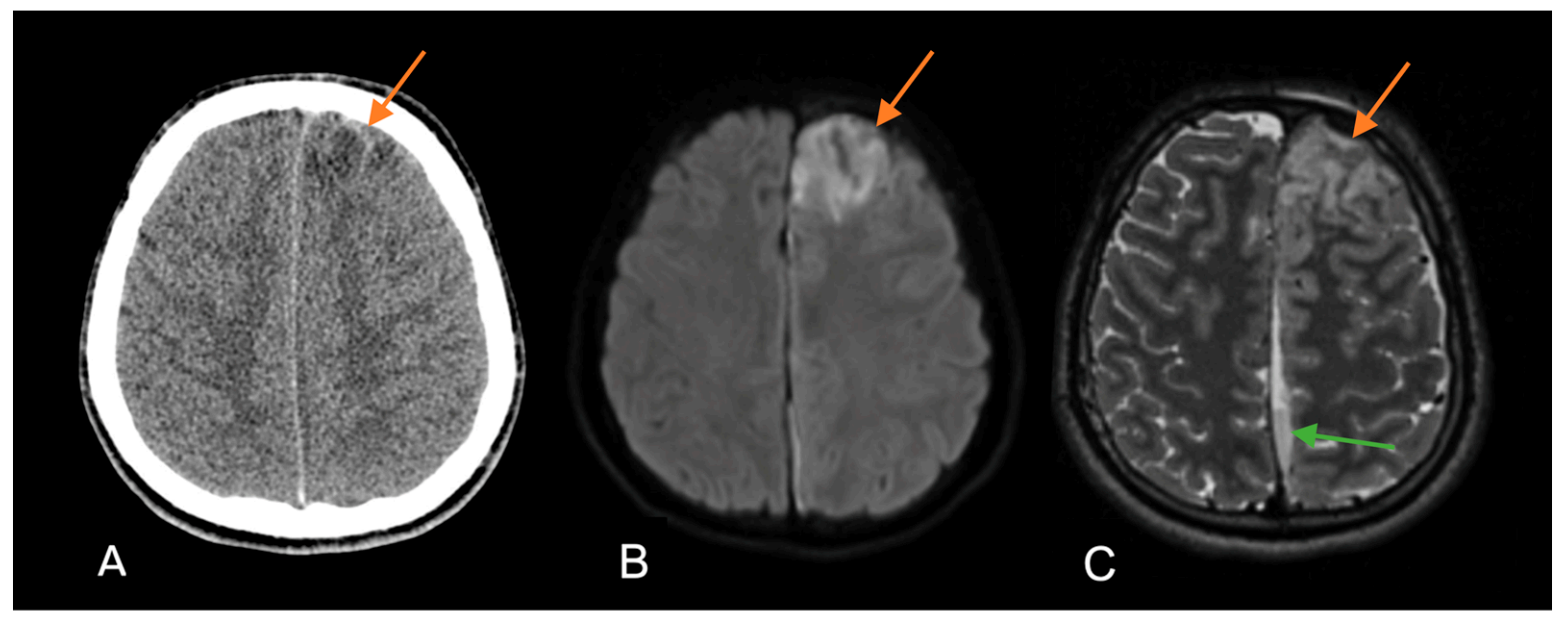
Initial cranial imaging. (**A**) Native cranial CT—the arrow shows a left frontal hypodense lesion with hemorrhagic components. (**B**) Axial diffusion-weighted MRI—the arrow shows left frontopolar cerebritis and restricted diffusion compatible with purulent intracranial infection. (**C**) Axial T2-weighted MRI—the orange arrow shows left frontal parenchymal hyperintensity and the green arrow shows an adjacent parafalcine/subdural empyema.

Native cranial CT demonstrated a left frontal hypodense lesion measuring approximately 35 × 27 mm with hemorrhagic transformation and a left frontal perfusion deficit. A small hypodense parafalcine rim was also described. The paranasal sinuses were partially opacified. MRI and MR angiography/phlebography performed the same day showed left frontopolar cerebritis with hemorrhagic components, left-sided intracranial empyema extending along the falx cerebri, infratentorial extension along the right tentorium, and intraspinal extension down to approximately C2. There was bilateral frontal, maxillary, and ethmoidal sinusitis with fluid levels. In the left frontal sinus region, subcutaneous frontopolar/supraorbital extension was suspected, consistent with empyema extension. Bihemispheric pachymeningeal enhancement was present. These findings, as shown in Figure 1, supported invasive sinogenic CNS infection rather than isolated viral illness or ischemia. Sinus vein thrombosis was ruled out.

The patient underwent emergency bilateral navigated pansinus surgery on the same day for presumed sinogenic meningitis with intracranial empyema. Intraoperatively, purulent sinusitis was confirmed bilaterally, more pronounced on the left. Increased milky-purulent secretion was encountered in the left frontal sinus. Both maxillary sinuses, ethmoid cells, and frontal sinuses were surgically opened and treated. Histopathology of the paranasal sinus tissue later showed chronic and florid inflammatory sinus mucosal changes, including partially purulent sinusitis. An external ventricular drainage was placed on the right side for intracranial pressure monitoring. The cerebrospinal fluid was cloudy. The initial intracranial pressure was initially documented as 5–7 mmHg.

Microscopy of the blood cultures on day 1 demonstrated anaerobic Gram-negative rods. Given the sinogenic origin of the infection and the possibility of a polymicrobial infection, antimicrobial therapy was escalated on day 1 postoperatively in the intensive care unit from ceftriaxone to the broader-spectrum antibiotic meropenem, administered as a continuous intravenous infusion at a total daily dose of 6 g. Acyclovir and Dexamethasone were then discontinued. The detection of *Klebsiella aerogenes* in the nasal swab further supported discontinuation of ceftriaxone, since this AmpC-producing organism may develop resistance during therapy with third-generation cephalosporins. On day 3, *F. necrophorum* was identified by MALDI-TOF mass spectrometry with scores > 2.2 from blood cultures, cultures of purulent material from the paranasal sinuses, and parafalcine swabs. Identification was additionally supported by typical growth under anaerobic culture conditions and compatible Gram-stain morphology. Antimicrobial susceptibility testing was performed by agar diffusion according to EUCAST and demonstrated susceptibility to penicillin G, piperacillin/tazobactam, meropenem, clindamycin, and metronidazole. Meropenem was therefore continued, and additional metronidazole was not considered necessary. The concordant detection of *F. necrophorum* in blood and operative specimens confirmed the organism as the pathogen of the invasive intracranial infection. Influenza-specific antiviral therapy was not administered.

During the early postoperative course, the patient developed increasing intracranial pressure and progressive mass effect. On hospital day 2, emergency left hemicraniectomy with evacuation of subdural and parafalcine empyema was performed. Intraoperatively, pus drained from the region of the left frontal sinus, supporting extension from the sinus infection. After dural opening, massive putrid secretion was evacuated from the subdural and interhemispheric/parafalcine regions, and additional purulence was released around the skull base and olfactory groove. The brain was markedly swollen.

Later the same day, follow-up CT demonstrated an enlargement of the left subdural empyema, with maximal thickness increasing from 3 to 12 mm, and of the parafalcine empyema, increasing from 7 to 12.6 mm, accompanied by progressive extracranial cerebral herniation through the hemicraniectomy defect. During reoperation large amounts of pressurized purulent material were evacuated from the skull base, sphenoid wing, and interhemispheric regions. The left frontal abscess area was opened and drained and an irrigation drain was placed.

**Figure 2:**
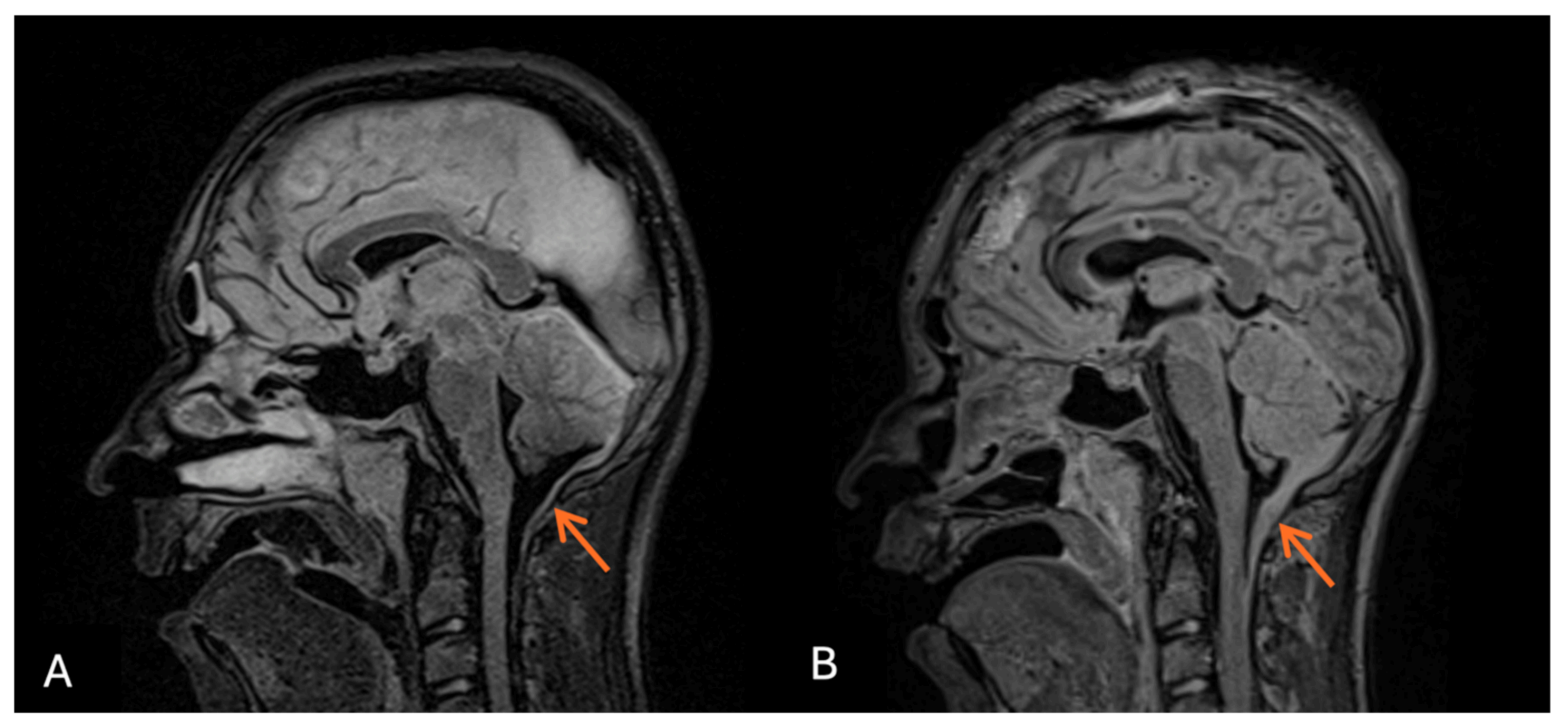
Sagittal FLAIR MRI on hospital day 1 (**A**) and hospital day 3 (**B**) demonstrating marked infratentorial progression of the empyema. The arrows in (**A**,**B**) show the progression of the extra-axial empyema measuring up to 7.7 mm extended along the midline and dorsally into the spinal canal.

On hospital day 3, MRI confirmed complete evacuation of the parafalcine empyema but demonstrated marked infratentorial progression, as demonstrated in Figure 2. Extra-axial empyema measuring up to 7.7 mm in thickness extended along the midline and dorsally into the spinal canal. Emergency suboccipital craniectomy with C1 laminectomy and evacuation of the subdural empyema was therefore performed. Intraoperatively, viscous purulent material was seen in the subarachnoid compartment. Later posterior fossa cultures remained negative after prolonged incubation, in the context of broad antibiotic pretreatment.

Further ENT Examination after detection of *F. necrophorum* showed no evidence of tonsillitis or pharyngeal focus. Examination by our maxillofacial surgeons showed no odontogenic focus.

The systemic inflammatory response remained severe throughout the clinical course. C-reactive protein increased from 272.9 mg/L on hospital day 1 to 470.0 mg/L on day 2, followed by 331.0 mg/L on day 3 and 304.5 mg/L on day 4. Leukocytes increased from 12.8 G/L on admission to 39.8 G/L on day 2, 30.6 G/L on day 3, and 43.0 G/L on day 4. The leukocytosis was predominantly neutrophilic, with 98.2% neutrophils and marked bandemia of 26.6% on admission, reflecting a pronounced acute bacterial inflammatory response; neutrophilic predominance persisted on day 3 (95.0%). Procalcitonin increased from 25.0 µg/L on day 1 to >100 µg/L on day 2 and remained >100 µg/L on day 4. The admission complete blood count showed a hemoglobin level of 114 g/L and a platelet count of 252 G/L; by day 3, hemoglobin had decreased to 77 g/L and platelets to 108 G/L. Biochemical deterioration became particularly pronounced on day 4, with hypoalbuminemia (20 g/L), acute renal dysfunction (creatinine 1.71 mg/dL), severe hyperkalemia (7.3 mmol/L), and marked increases in AST (2877 U/L), ALT (530 U/L), LDH (4409 U/L), and creatine kinase (159,141 U/L), consistent with progressive septic multiorgan failure.

**Figure 3:**
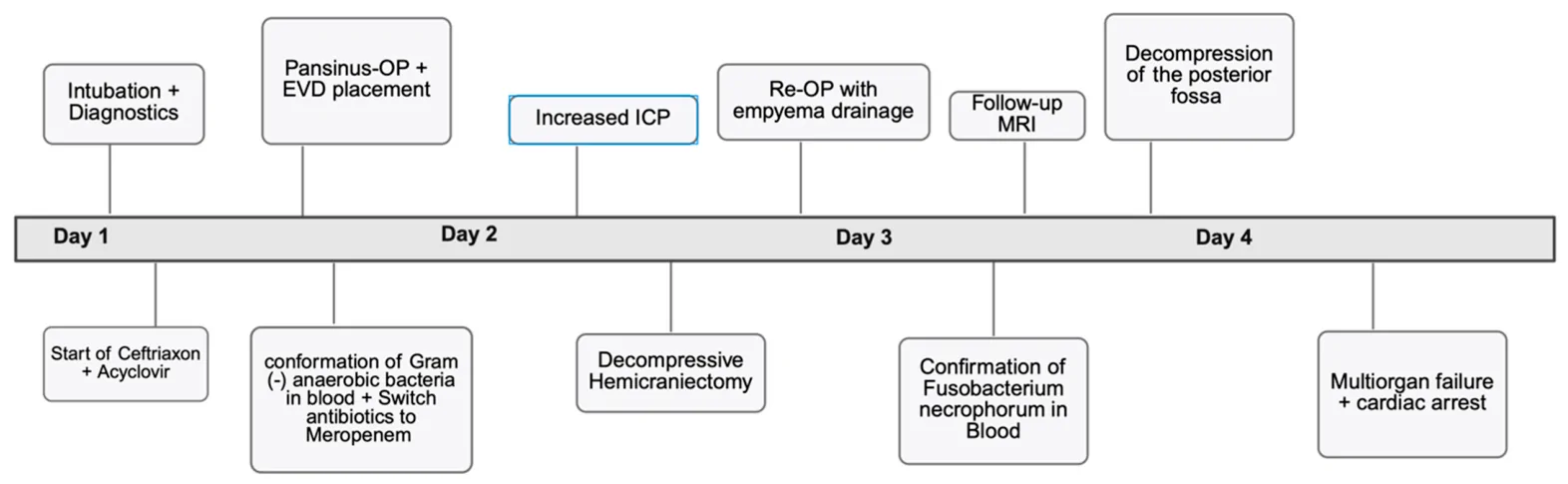
Clinical timeline of the disease course from hospital day 1 to 4, showing key events. The indication for decompressive hemicraniectomy was based on the increased ICP as showed in the blue box.

In the early morning of day 4 of hospital stay, progressive hyperkalemia prompted the start of dialysis. Before renal replacement therapy could be started, the patient developed QT prolongation, widening QRS complexes, new left bundle branch block, bradycardia, and ultimately asystole. Resuscitation included repeated calcium gluconate, glucose/insulin, sodium bicarbonate, and continuous adrenaline infusion. Bedside echocardiography during CPR showed a thrombus in the left ventricle. Following interdisciplinary consensus among Neurology, Neurosurgery, Cardiology, and Anesthesiology, resuscitative efforts were discontinued after approximately 30 min. Figure 3 illustrates the key clinical events.

A subsequent neuropathological examination of left parafalcine abscess material demonstrated purulent leptomeningoencephalitis with dense neutrophilic leptomeningeal infiltrates extending into Virchow–Robin spaces and brain parenchyma, pronounced fresh cortical microhemorrhages, and interestingly isolated Gram-positive coccoid bacteria on Gram stain, suggesting a polymicrobial infection.

## 3. Discussion

This case illustrates the fulminant end of the clinical spectrum of *Fusobacterium necrophorum* central nervous system infection. The patient was a previously healthy young adult who initially presented with a severe febrile illness, sinusitis, meningismus, altered mental status, rapidly progressive intracranial infection and a positive *Influenza A* test. Despite early broad antimicrobial therapy and emergency surgery, the disease progressed to extensive septic multiorgan failure and death within four hospital days.

The distinctive feature of this case is the exceptionally rapid and extensive progression of a sinogenic *F. necrophorum* infection, with supra- and infratentorial empyema, spinal extension, cerebritis, and fatal septic multiorgan failure within four hospital days. Although similar complications have been reported individually, this combination represents an unusually fulminant presentation in a previously healthy young adult. Concomitant *Influenza A* infection further distinguishes the case, although its contribution remains uncertain.

The clinical course of reported *F. necrophorum* CNS infections is heterogeneous. Somoza et al. described a previously healthy 20-year-old man with influenza followed by persistent sinusitis symptoms and a monomicrobial *F. necrophorum* intracranial abscess arising from right frontal sinusitis; despite recurrence requiring repeat craniotomy and antibiotic escalation, the patient ultimately recovered [11]. In contrast, Sahhar et al. reported an immunocompetent 17-year-old with sinusitis-associated subdural empyema caused by *F. necrophorum* and *Arcanobacterium haemolyticum*, who developed extensive infarction, cerebral edema, herniation, and brain death despite emergent decompressive hemicraniectomy [8].

Other reports show that *F. necrophorum* CNS disease is not limited to sinogenic spread. Otitis media and mastoiditis are repeatedly described as major sources, particularly in children [2,3,4,6,12,13,14,15,16,17]. Feenstra et al. reviewed pediatric *F. necrophorum* meningitis and found two apparent age clusters: preschool children, in whom otitis media or mastoiditis predominated, and adolescents, in whom sources were more diverse, including sinusitis and peritonsillar infection [6]. Gingival or oral infection may also serve as the primary focus, as shown by Duquesne et al., who reported fulminant meningoventriculitis in an 11-month-old infant with gingivitis and fatal outcome within 72 h [18]. Rarely, a clear contiguous source is not identified, and hematogenous spread has been proposed, particularly when abscesses are distant from the meninges or when bony destruction is absent [12,13,16]. In our patient, the combination of imaging findings, purulent frontal sinusitis, intraoperative drainage from the frontal sinus region, and exclusion of tonsillar, pharyngeal, odontogenic, and otogenic sources supports frontal sinusitis as the origin.

The pathophysiology appears to differ according to the source. Otogenic cases may spread through direct invasion, thrombophlebitis, or hematogenous dissemination; several reports noted intact bony structures despite severe intracranial disease, suggesting that visible bone destruction is not required for CNS spread [12,13,16]. In sinogenic cases, contiguous intracranial extension from the frontal or paranasal sinuses appears more plausible, especially when imaging and operative findings show direct anatomical continuity [8,11]. In Lemierre-type disease, the classical sequence involves oropharyngeal infection, septic thrombophlebitis, and metastatic spread, whereas atypical presentations may include meningeal or cerebral involvement without the full classical syndrome [4,18]. The pronounced tendency toward thrombosis and infarction in several cases has been attributed to virulence factors such as endotoxins, leukotoxins, hemolysins, hemagglutinins, and coagulase-like activity, which may promote local anaerobic infection, platelet aggregation, venous thrombosis, and systemic coagulopathy [4,13,18].

A major diagnostic lesson from the literature is that *F. necrophorum* CNS infection may be missed if anaerobic microbiology is not specifically pursued. Several cases had negative routine CSF or local cultures, while diagnosis was made through anaerobic blood culture, cytocentrifuged CSF microscopy, specialized anaerobic media, MALDI-TOF, sequencing, or operative abscess culture [2,15,16,19]. Tärnvik et al. showed that ordinary CSF culture plates may remain negative while an anaerobic blood-culture-type medium grows *F. necrophorum* [15]. Angelino et al. similarly reported negative CSF despite positive anaerobic blood cultures [2]. This is directly relevant to our case: CSF culture and molecular testing remained negative after ceftriaxone pretreatment, whereas anaerobic blood cultures and multiple operative intracranial samples established *F. necrophorum* as the pathogen.

Treatment in reported cases generally combined antimicrobial therapy and source control. Metronidazole is frequently emphasized because of its reliable anaerobic activity and CNS penetration, and it has been used in combination with third-generation cephalosporins, penicillin-based regimens, beta-lactam/beta-lactamase inhibitor therapy, clindamycin, meropenem, or other broad-spectrum agents depending on disease severity, suspected polymicrobial infection, and susceptibility data [2,3,6,13,17,18]. In severe intracranial suppurative disease, meropenem represents a rational broad-spectrum option, providing bactericidal activity against anaerobic Gram-negative rods including Fusobacterium spp. while maintaining coverage for relevant aerobic pathogens that would not be covered by metronidazole alone [20,21]. In our patient, the decision to use meropenem rather than continuing ceftriaxone with additional metronidazole was based on the sinogenic and potentially polymicrobial origin of the infection. At the time of escalation, only Gram-negative rods had been detected in blood cultures and the causative organism had not yet been identified. Furthermore, *Klebsiella aerogenes* had been detected in a nasal swab. As an AmpC-producing organism, it may develop resistance during treatment with third-generation cephalosporins, making continued ceftriaxone therapy less favorable. Meropenem therefore provided broader coverage of anaerobic organisms as well as potentially relevant sinus pathogens, including *streptococci* and the detected *K. aerogenes*. After *F. necrophorum* was identified and susceptibility to meropenem was confirmed, additional metronidazole was not considered necessary. This strategy is consistent with contemporary management principles for intracranial suppurative infections, which emphasize early broad empirical therapy with adequate CNS penetration, neurosurgical source control when indicated, and prolonged intravenous treatment [10]. Several reports also caution that in-vitro susceptibility may not reliably predict clinical response and that short or insufficient therapy may be followed by relapse [13,15,16,17].

Surgical management varies with anatomy and disease extent: reported interventions include tympanostomy, mastoidectomy, sinus drainage, craniotomy for empyema or abscess evacuation, decompressive craniectomy, and external ventricular drainage [3,4,6,8,11,14,16].

The prognosis of *F. necrophorum* CNS infection remains guarded. Feenstra et al. reported that among pediatric cases with available outcome, a substantial proportion either died or survived with neurological morbidity [6]. Older reviews and fatal case reports similarly describe mortality around one-third and frequent neurological deficit among survivors, particularly cranial nerve palsies, hemiparesis, infarction-related deficits, or severe motor impairment [3,4,13]. Importantly, fatal progression may occur even after antimicrobial escalation and laboratory improvement, as shown by Angelino et al., where metronidazole improved inflammatory parameters but could not reverse established intracranial hypertension and herniation [2]. Our case similarly suggests that once diffuse empyema, cerebritis, posterior fossa involvement, and septic systemic deterioration are established, aggressive treatment may not prevent fatal outcome.

Lastly, the possible role of concomitant *Influenza A* infection warrants consideration. Influenza infection may increase susceptibility to secondary bacterial infection through alterations of innate and adaptive immune responses. In particular, impaired macrophage and neutrophil-mediated bacterial killing, reduced antimicrobial peptide activity, and interferon-mediated suppression of antibacterial Type 17 immunity have been described [22]. Influenza-associated epithelial injury and impairment of normal respiratory clearance mechanisms may further facilitate bacterial invasion from the upper respiratory tract [23]. In our patient, these mechanisms may have facilitated invasion by *F. necrophorum* and contributed to the unusually rapid and severe disease course. Clinical observations supporting a possible association include a reported case of *F. necrophorum* Lemierre’s syndrome following *Influenza A* infection [24] and a case of *F. necrophorum* pneumonia with concomitant Influenza A infection [23]. However, evidence specifically linking *Influenza A* to invasive *F. necrophorum* infection remains very limited, and its contribution to the course of our patient therefore remains speculative.

## 4. Conclusions

This case highlights *Fusobacterium necrophorum* as a rare but highly aggressive anaerobic pathogen capable of causing fulminant sinogenic CNS infection in otherwise healthy young adults. Diagnostic workup should not rely on CSF studies alone, as cultures and molecular tests may remain negative after early antibiotic exposure; anaerobic blood cultures and operative intracranial specimens were decisive in our case. Early consideration of anaerobic pathogens and appropriate anaerobic culture techniques are therefore essential in severe sinogenic CNS infections. Management requires prompt broad-spectrum antimicrobial therapy together with early multidisciplinary source control involving neurosurgical, otorhinolaryngological, microbiological, and intensive care expertise. Despite high-dose meropenem and repeated surgical intervention, our patient developed extensive supra- and infratentorial empyema, cerebritis, spinal spread, and fatal septic multiorgan failure within four hospital days. This case emphasizes the importance of early anaerobic diagnostics, rapid interdisciplinary management, and aggressive source control in similarly fulminant presentations.

## Data Availability

The data generated and analyzed during this study are not publicly available due to patient privacy and ethical restrictions. De-identified data may be available from the corresponding author on reasonable request, subject to institutional review and approval.
